# The role of oxytocin in familiarization-habituation responses to social novelty

**DOI:** 10.3389/fpsyg.2013.00761

**Published:** 2013-10-18

**Authors:** Mattie Tops, Renske Huffmeijer, Mariëlle Linting, Karen M. Grewen, Kathleen C. Light, Sander L. Koole, Marian J. Bakermans-Kranenburg, Marinus H. van IJzendoorn

**Affiliations:** ^1^Centre for Child and Family Studies, Leiden UniversityLeiden, Netherlands; ^2^Leiden Institute for Brain and Cognition, Leiden UniversityLeiden, Netherlands; ^3^Department of Clinical Psychology, VU University AmsterdamAmsterdam, Netherlands; ^4^Department of Psychiatry, University of North CarolinaChapel Hill, NC, USA; ^5^Department of Anesthesia, University of UtahSalt Lake City, UT, USA

**Keywords:** oxytocin, trust, stress habituation, novelty, familiarity, familiarization-habituation response

## Abstract

Stress or arousal responses to novel social contexts ease off when individuals get familiar with the social context. In the present study we investigated whether oxytocin is involved in this process of familiarization-habituation as oxytocin is known to increase trust and decrease anxiety. Fifty-nine healthy female subjects took part in the same experimental procedure in two sessions separated by 4 weeks. In the first (novelty) session state trust scores were significantly positively correlated with salivary oxytocin levels while in the second (familiarity) session state trust scores were significantly negatively correlated with salivary oxytocin levels. In a path model oxytocin was associated with increased trust in the novelty session and trust was associated with decreased oxytocin levels in the familiarity session. The results are consistent with the idea that oxytocin decreases stress-to-novelty responses by promoting familiarization to novel social contexts.

## Introduction

Stress and arousal responses to novel social contexts habituate when individuals get familiar with the social context (e.g., Tops et al., 2006a; Balodis et al., 2010). Building on the literature on the neuropeptide oxytocin and stress we proposed that oxytocin facilitates this process through a “familiarization-habituation response” (Tops et al., 2013a,b). In the present study, we consider how this stress-habituation process is related to the peripheral levels of oxytocin.

A review of the past 60 years of human oxytocin research concluded that oxytocin reduces fear and induces feelings of calm and trust, as well as endocrine and physiological changes (Ishak et al., 2011). A central function of oxytocin may be to facilitate social encounters in novel environments and with unfamiliar others by reducing uneasiness and anxiety (McCarthy, 1995). Indeed, anxiolytic effects of oxytocin have been demonstrated in a variety of species (Carter, 1998; Uvnäs-Moberg et al., 2005). These effects occur both after exogenous oxytocin administration and after endogenous release (Neumann, 2008). Oxytocin may facilitate habituation of stress responses. In other words, oxytocin may enable familiarization-habituation responses to stress as implied in social novelty. Oxytocin is, for example, released in challenging situations that trigger coping efforts and anxiety, including challenges of social evaluative stress (Pierrehumbert et al., 2010; Jezova et al., 2013). Furthermore, social support following intense psychological stress can promote the release of oxytocin while attenuating the physiological and behavioral stress response toward a subsequent stressor in pair-bonded female prairie voles (Smith and Wang, 2012). In humans, social support increases peripheral oxytocin, attenuates the cortisol response and increases calmness during psychosocial stress (Grewen et al., 2005). Intranasal oxytocin treatments can potentiate these calming effects (Heinrichs et al., 2003; Quirin et al., 2011).

Oxytocin may thus be functionally involved in the habituation to social challenge and novelty and, hence, oxytocin responses may occur only as long as there has not been sufficient habituation to the social stimulus or context (Danevova et al., 2013). Illustrating this point, it was found that young women who recently experienced a social conflict showed an oxytocin response to a simulated confrontation with the transgressor (Tabak et al., 2011). Although there was no mean increase in oxytocin over all participants, a larger oxytocin response to the confrontation was associated with low calmness and trust toward the transgressor (measured immediately after the confrontation) and low forgiveness of the transgressor. The correlations of the oxytocin response to low calmness, trust and forgiveness suggest an association with an unresolved conflict, responses to which did not yet habituate. Similarly, Bick and Dozier (2010) found that mothers displayed higher oxytocin levels when familiarizing themselves with previously unknown children than when interacting with their own children. Additional evidence that oxytocin responses are associated with coping attempts that require formation of trust, which in turn facilitates social stress habituation, has been provided by Kéri and Kiss (2011). They showed that a social challenge requiring trust, but not a cognitive challenge, was associated with an increase in oxytocin levels. Moreover, only the oxytocin response to the trust challenge was associated with efficient habituation of physiological arousal (Kéri and Kiss, 2011; Kiss et al., 2011). As a last example, intranasal oxytocin increased self-perceived trust only in participants who reported negative mood after social rejection, which was interpreted as motivating social support seeking (Cardoso et al., 2013).

Recent intranasal oxytocin application studies demonstrated the involvement of oxytocin in interpersonal trust and cooperation (see Bartz et al., 2011). For instance, when subjects interacted with strangers, intranasal oxytocin reduced the fear of being exploited and betrayed (Baumgartner et al., 2008) and increased trust (Kosfeld et al., 2005). Intranasal oxytocin also increased self-rated social trust, warmth and friendliness (Cardoso et al., 2012). Underscoring the importance of familiarization/habituation, oxytocin increased cooperation only when participants familiarized themselves with their interaction partner (Declerck et al., 2010), when protagonists were described as trustworthy (Mikolajczak et al., 2010), or when protagonists belonged to one's in-group (De Dreu et al., 2010). In turn, higher trust predicts lower social stress responses (Takahashi et al., 2005). Trust may be a social-specific elaboration of more general mechanisms of habituation and familiarization. We suggest that oxytocin increases sensitivity to socially rewarding stimuli signaling that the social environment allows for increases in trust.

Prior research has examined the familiarization-habituation process using a two-session within-subject design. A typical finding is that both positive and negative affect increased when subjects were introduced to a novel social context at the start of the first session, habituated during the course of this session, and they were lower during a second session (Wirth et al., 2011; cf. Tops and Wijers, 2011). Similarly, hormonal mediators of stress coping (cortisol and alpha-amylase) increased at first arrival and habituated during the first session (Tops et al., 2006a; Balodis et al., 2010). Moreover, individuals showing higher levels at arrival also show larger decreases during the session (Tops et al., 2006a) and larger hormonal and subjective anxiety responses to acute stressors (Balodis et al., 2010), which implies that high levels at arrival reflect a stress coping response. The increase in both positive and negative affect, as well as indices of hormonal stress likely reflects an active coping with a novel social context (Cardoso et al., 2013). These affective and hormonal indicators of stress may become down-regulated (i.e., habituate) when the social context turns out to be sufficiently benign. As explained above, it seems plausible that this familiarization-habituation response is facilitated by oxytocin (see also Tops et al., 2007a; Campbell, 2008).

According to the familiarization-habituation hypothesis, active social coping responses to a novel but permissive context are associated with elevated oxytocin levels and trust, producing a positive correlation between oxytocin and trust. Moreover, subjects who show increased trust no longer show elevated oxytocin when encountering the context a second time. However, subjects who do not show increased trust (which we assume is related to smaller elevation of oxytocin at the first encounter) still need to cope and therefore still show elevated oxytocin at the second encounter. Although the subjects who do not show increased trust show smaller elevation of oxytocin, the small elevation of oxytocin in those subjects relative to no elevation in the subjects who showed increased trust produces a negative correlation between trust and oxytocin at the second encounter. In other words, higher oxytocin levels may be associated with faster / greater familiarization. On the other hand, higher familiarity and trust lead to lower levels of oxytocin.

In the present study, we investigated the role of oxytocin in the familiarization-habituation process within a larger study focusing primarily on the effects of experimentally manipulated oxytocin on event-related potentials (ERPs; see Huffmeijer et al., 2013, for a report). As part of this ERP research, participants completed two identical experimental sessions that were scheduled 4 weeks apart. During each session, we measured participants' baseline salivary oxytocin levels (i.e., before oxytocin administration) along with participants' experienced state trust, allowing us to examine how naturally occurring oxytocin relates to the familiarization-habituation response. Specifically, we expected that exposure to the first experimental session (the “novelty session”) would lead participants to experience an increase in feelings of uneasiness, which would lead to an increase in oxytocin levels, which in turn would be associated with higher levels of trust. Furthermore, we expected that individuals who showed higher oxytocin and trust during the novelty session would become habituated to the situation. Higher trust might thus be associated with a decrease in oxytocin levels from the novelty session to the second session (the “familiarity session”). In contrast, individuals who display lower trust may show reduced habituation, because lower social trust has been associated with decreased stress response (e.g., cortisol) habituation (Kirschbaum et al., 1995; Pruessner et al., 1997).

## Materials and methods

### Participants

A total of 59 females undergraduate students took part in the experiment. They were paid 50 Euros for participation. Two participants completed only the first session. The final sample thus consisted of 57 participants (aged 18–30 years, *M* = 20.51, *SD* = 2.90). Exclusion criteria were colorblindness, smoking, alcohol and drug abuse, neurological and psychiatric disorders, pregnancy, breastfeeding, and use of medication (except oral contraceptives, which was recorded as a background variable). The study was approved by the research ethics committee of the Leiden University Medical Center.

### Design and procedure

Participants were asked to come to our laboratory for two identical experimental sessions, separated by approximately 4 weeks, that included, among other assessments, an ERP experiment (Huffmeijer et al., 2013) with double-blind administration of oxytocin and placebo. The effects of nasal oxytocin application are reported elsewhere (Huffmeijer et al., 2012, 2013). In the present article, we present our findings for endogenous oxytocin levels in saliva collected before spray application of each session. To minimize influences of diurnal variations in oxytocin levels, all sessions took place in the afternoon (starting between 12.00 and 15.00). Participants were instructed to abstain from alcohol and excessive physical activity during the 24 h before the start of each session, and from caffeine on the day the session took place. Informed consent was obtained at the beginning of the first session. At the start of each session (t1), a saliva sample was collected and participants self-reported state trust, as well as calmness and uneasiness. Participants were then fitted with an electrode net after which they completed an Eriksen flanker task (Eriksen and Eriksen, 1974), with a short break halfway through. Participants' EEG was acquired during performance of the flanker task using 129-channel hydrocel geodesic sensor nets. The analyses in the present paper only include the t1 measures. Halfway through (approximately 1¼ h after t1) and after completion of an ERP experiment (approximately 2¼ h after t1) saliva samples were collected and participants again self-reported mood states.

The stressor–which we hypothesized would affect trust, calmness, and uneasiness–consisted of social and non-social aspects of the context of the experimental session. This context included introduction to and interaction with the unfamiliar experimenter, social uneasiness resulting from collecting saliva in the presence of the experimenter, a certain level of intimacy because electrode application requires some physical contact, and task performance. Task performance that is not associated with personal outcomes such as academic success, is mainly stressful because it triggers social evaluative concerns (Dickerson and Kemeny, 2004). Even non-social aspects of the novel context may trigger social coping such as responsivity to, or elicitation of, reassurance from the experimenter. In the present study, session 1 (the novelty session) and session 2 (the familiarity session) were identical in variables including the identity of the experimenter, the location, time of day, tasks, procedures, and measures.

### State trust

To measure the state intensity of trust we adapted the Carstenson Emotion Questionnaire (CEQ; Dywan et al., 2008). The CEQ was adapted by Dywan et al. (2008) to a measure of the intensity in which emotions are experienced. In the present study, the participants were asked to rate how intensely they experienced trust, calmness and uneasiness during the previous 10 min on 7-point rating scales (1 = “not very intense,” 7 = “very intense”).

### Salivary oxytocin

For each sample at least 1 mL of unstimulated saliva was collected into 1.8 mL cryotubes using the passive drool method. Samples were immediately frozen and were stored at −20° Centigrade until batch assay. Level of oxytocin in saliva was assayed using a commercially available kit as per the method previously described (Holt-Lunstad et al., 2008; Grewen et al., 2010). Prior to the enzyme immunoassay procedure, in keeping with the manufacturer's strong recommendation, an extraction step was performed based on instructions accompanying the EIA kit currently available in February 2011 (ADI-900-153, Enzo Life Science, Plymouth Meeting, PA). The result of this extraction was to concentrate the sample 3.2 times, increase precision and reduce matrix interference. Oxytocin extraction efficiency was 93%, which was determined by spiking with a known amount of hormone and extracting this known amount along with the other samples. Oxytocin levels in extracted saliva were then quantified using the OT EIA, in which the endogenous oxytocin hormone competes with added oxytocin linked to alkaline phosphatase for oxytocin antibody binding sites. After overnight incubation at 4°C., the excess reagents were washed away and the bound oxytocin phosphatase was incubated with substrate. After 1 h this enzyme reaction, which generates a yellow color, was stopped and the optical density (OD) was read on a Sunrise plate reader (Tecan, Research Triangle Park, NC). The intensity of the color at 405 nm is inversely proportional to the concentration of oxytocin. The hormone content (in pg/mL) was determined by plotting the intensity of OD of each sample against a standard curve. Following correction for extraction, the lower limit of sensitivity was 1.25 pg/mL. Less than 1% of the samples fell below the lower level of sensitivity (4 out of 348). These values were subsequently replaced with the lowest detectable level of 1.25 pg/mL. The intra- and inter-assay coefficients of variation were 7.35 and 8.51% respectively. The manufacturer has reported that cross-reactivity with similar mammalian neuropeptides is less than 1%.

For one participant, one oxytocin value was considered an outlier (*z* > 3.29) within its time point (t1) and condition. For statistical analysis, this value was replaced with the highest value occurring at that time point and condition among the remaining participants. To normalize data distribution, we computed the natural logarithm of the raw values.

### Statistical analyses

#### Main analyses

All analyses were performed on baseline trust and endogenous oxytocin levels before intranasal oxytocin application. The associations between self-reported trust and oxytocin levels were computed with Pearson correlations. State trust and oxytocin levels were analyzed using General Linear Model (GLM) analyses with session (novelty vs. familiarity) as within subject factor. Partial eta squared (η^2^_*p*_) is reported as measure of effect size. We estimated a path model using EQS (Bentler, 1995) to test whether the data were consistent with our hypothesis that oxytocin in the novelty session predicts higher state trust during that session, which in turn predicts trust in the familiarity session, lowering oxytocin in the familiarity session.

#### Control analyses

Analyses were also performed including use of oral contraceptives, phase of menstrual cycle, age and order of drug administration as covariates. We show in the Results section that these variables did not influence outcomes of the analyses, and we report subsequent analyses without these covariates.

Intranasal oxytocin application effects are not the focus of the present analyses, but it is conceivable that these applications may have impacted upon state trust and for those subjects who received oxytocin in the first session, effects may have carried over to trust scores or salivary oxytocin responses at t1 in the second session. Although a GLM analysis of state trust with condition (oxytocin or placebo) and time point as within subject factors and order of administration (placebo first or oxytocin first) as between subjects factor, did not show trends for main effects of condition or interactions with time, we present additional control analyses that rule out effects of intranasal oxytocin. There were two orders of intranasal oxytocin administration. In order 1, the subjects received placebo in the first session and oxytocin in the second session. In order 2 this was the other way around. As we use only the oxytocin levels and the trust scores before nasal oxytocin application, in order 1 all measurements were obtained before the subjects received any oxytocin, eliminating any possibility of confounding by intranasal oxytocin. We present the result of this analysis in the Results section. Significance of the effect in order 1, along with similar results obtained in order 2 would show that the results are not confounded by nasal oxytocin application. Moreover, this split-half analysis of similar results in two independent randomized sub-groups tests the reliability of the results.

To check the validity of our trust and oxytocin measures and the interpretation of effects, we investigated whether they related to changes in state calmness and uneasiness that suggest increased habituation over sessions.

## Results

### Main analyses

**Figure 1:**
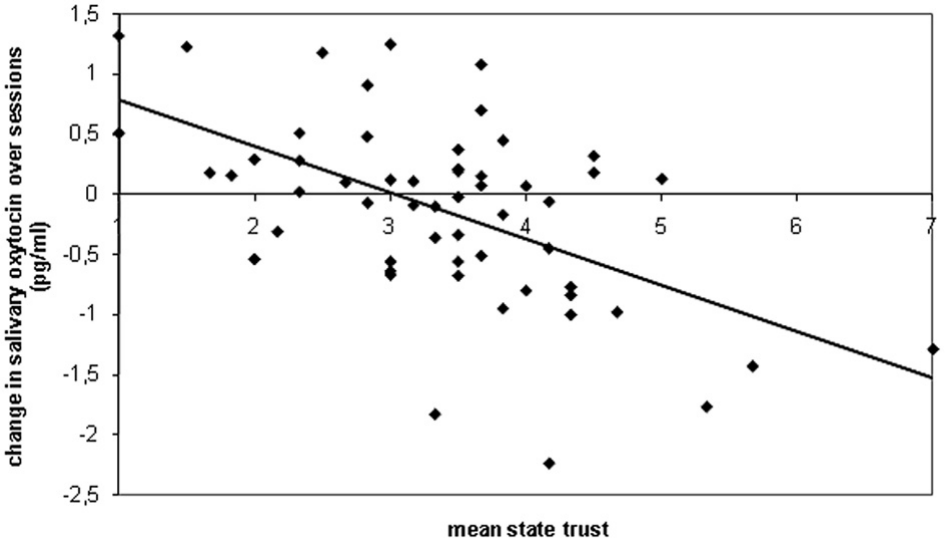
**Scatterplot of the relationship between trust scores (mean over both sessions) and the change in salivary oxytocin levels (familiarity session minus novelty session)**. If our hypothesized model (see Figure 2) is correct, than the same subjects who displayed high trust and oxytocin in the novelty session, showed high trust and low oxytocin in the familiarity session. This pattern may produce an association between the mean trust over sessions and the decrease in oxytocin over sessions. If, on the other hand, contrary to our hypothesis, the positive correlation between trust and oxytocin in the novelty session is not displayed by the same subset of subjects that displayed a negative correlation between trust and oxytocin in the familiarity session, then a correlation between mean trust and change in oxytocin is unlikely. Consistent with our hypothesis, the scatterplot shows that the mean trust score correlated with a larger decrease in oxytocin levels over sessions.

Trust was higher in the novelty session (*M* = 4.32, *SD* = 1.51) than in the familiarity session [*M* = 3.81, *SD* = 1.51; *F*_(1, 56)_ = 5.72, *p* = 0.020, η^2^_*p*_ = 0.093]. Trust correlated positively to salivary oxytocin level in the novelty session (*r* = 0.29, *p* = 0.031), but correlated negatively to salivary oxytocin in the familiarity session (*r* = −0.31, *p* = 0.019). Including the mean state trust over sessions (i.e., the mean of the session 1 and the session 2 score) as a continuous predictor in the GLM analysis of oxytocin levels showed that higher mean state trust was associated with a decrease in oxytocin from the novelty (*M* = 1.91 pg/mL, *SD* = 0.62) to the familiarity session [*M* = 1.78, *SD* = 0.58; *r* = −0.55, *F*_(1, 55)_ = 24.26, *p* < 0.001, η^2^_*p*_ = 0.306; see Figure 1]. This pattern is consistent with the interpretation that (possibly oxytocin-induced) increased trust leads to lower oxytocin response in the familiarity session. Inclusion as covariates of the control variables, use of oral contraceptives, phase of menstrual cycle, age and order of drug administration, only slightly increased the association between state trust and the change in oxytocin from the novelty to the familiarity session (partial *r* = −0.59, *p* < 0.001). The raw (non-transformed) oxytocin levels were 8.18 pg/mL (*SD* = 5.36) and 6.87 (*SD* = 3.75) in the novelty and familiarity session, respectively.

### Split-half reliability and ruling out intranasal oxytocin effects

As explained above, the subjects were randomly allocated to two orders of intranasal oxytocin administration. In order 1, the subjects received placebo in the first session and oxytocin in the second session. In order 2 this was the other way around. In order 1, the correlation between mean trust scores at t1 and the change in oxytocin level over sessions was *r* = −0.40, *p* = 0.040. In order 2 this correlation is *r* = −0.52, *p* = 0.003. If we aggregate the trust scores over the three measurement points within sessions, the correlations are *r* = −0.56, *p* = 0.002 and *r* = −0.55, *p* = 0.002, in order 1 and 2 respectively.

### Path model

**Figure 2:**
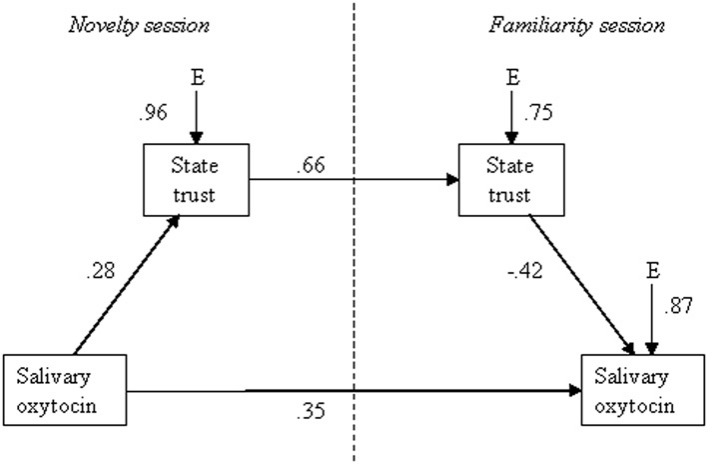
**Path diagram showing standardized path coefficients (*p*s < 0.05)**.

We estimated a path model using EQS (Bentler, 1995) to explore whether the data are consistent with our hypothesis that oxytocin in the novelty session positively influences state trust, which in turn increases trust in the familiarity session, which in turn lowers oxytocin in the familiarity session. To account for individual differences in basal oxytocin levels and in stress responses other than successful familiarization-habituation we also included the direct path from oxytocin level in the novelty session to oxytocin level in the familiarity session. There were no problems with non-normality or missing data. Figure 2 shows the path diagram. All paths in the model are significant in the expected direction (*p*s < 0.05). Despite a high root mean square error of approximation of 0.19, reasonable fit was indicated by indices of goodness of global approximation, Chi-square = 5.902, *df* = 2, *p* > 0.05 and by the comparative fit index of 0.92. As shown in Table 1, the covariance between oxytocin in the novelty session and trust in the familiarity session displayed the largest (still relatively small) residual. There was no indication that more parameters should be estimated or that parameters could be omitted (LMtest and Wtest, respectively). In conclusion, this statistical model showed reasonable fit with the data and with our theoretical model.

**Table 1:** **Correlations and residuals**.

	**Oxytocin N**	**Oxytocin F**	**Trust N**	**Trust F**
Oxytocin N		−0.074	0.0	0.173
Oxytocin F	0.210		−0.141	0.063
Trust N	0.290^*^	−0.320^*^		0.0
Trust F	0.350^**^	−0.310^*^	0.660^***^	

*Sample correlations are in lower triangle and residuals are in upper triangle. An exact zero is represented by 0.0. N, novelty session; F, familiarity session*.

* p < 0.05;

** p < 0.01;

*** p < 0.001.

### Validation check of the interpretation of measures and effects

To strengthen our case that our results reflect an association of oxytocin and trust with habituation over sessions, we checked whether, from the novelty to the familiarity session baselines, trust and decreased oxytocin were increasingly associated with calmness and decreasingly with uneasiness. Notably, both trust (see above) and uneasiness [novelty: *M* = 2.53, *SD* = 1.32: familiarity: *M* = 2.00, *SD* = 1.18; *F*_(1, 56)_ = 10.50, *p* = 0.002, η^2^_*p*_ = 0.158] were higher in the novelty session than the familiarity session, and both correlated positively with saliva oxytocin level in the novelty session (uneasiness: *r* = 0.38, *p* = 0.004). In the novelty session, higher trust showed a similar association with higher uneasiness as with higher calmness (*r* = 0.27 and *r* = 0.23, respectively), while in the familiarity session, higher trust correlated strongly with higher calmness (*r* = 0.51, *p* < 0.001) but not with uneasiness (*r* = 0.06, *n.s*.). Calmness decreased from the novelty session (*M* = 5.00, *SD* = 1.22) to the familiarity session [*M* = 4.58, *SD* = 1.22; *F*_(1, 56)_ = 4.34, *p* = 0.042, η^2^_*p*_ = 0.072] and correlated negatively to oxytocin level only in the familiarity session (*r* = −0.30, *p* = 0.022). Finally, including the difference in calmness between sessions as continuous predictor in a GLM analysis of oxytocin levels showed that oxytocin decreased from the novelty to the familiarity session [*F*_(1, 55)_ = 3.99, *p* = 0.049, η^2^_*p*_ = 0.068] and an increase in calmness predicted a decrease in oxytocin level [*r* = −0.31, *F*_(1, 55)_ = 5.87, *p* = 0.019, η^2^_*p*_ = 0.096]. This pattern of relationships is consistent with the hypothesis that oxytocin and trust are part of dynamical processes that differ between the novelty and the familiarity session: in the novelty session oxytocin and trust were associated with uneasiness and associated with, or increased by active social coping processes (Cardoso et al., 2013), while in the familiarity session trust reflected that social coping was successful and allowed for calmness and habituation.

## Discussion

State trust and oxytocin levels were higher at arrival in the novelty session than at arrival in the familiarity session. The decrease in oxytocin from the novelty session to the familiarity correlated with an increase in calmness. We suggest that in the novelty session trust was increased by active social coping processes as reflected in higher oxytocin levels, while in the familiarity session trust reflected that social coping was successful and allowed for calmness and habituation with decreased oxytocin levels. A path model was consistent with the theoretical model that oxytocin is associated with increased trust in the novelty session whereas trust decreased oxytocin levels in the familiarity session. It should be noted that our study was exploratory and needs to be replicated in independent samples. However, in accordance with our interpretation and theory (Tops et al., 2013a,b), previous studies have found evidence suggesting that positive and negative affect as well as hormonal mediators of stress coping are increased at arrival in a novel context in the first session of an experiment, habituate during that session, and are lower in a second session (Kirschbaum et al., 1995; Pruessner et al., 1997; Kudielka et al., 2006; Tops et al., 2006a; Balodis et al., 2010; Wirth et al., 2011). Higher levels of positive and negative affect and of hormonal mediators of stress coping at arrival in a novel social context may reflect active coping responses that are aimed at acclimatization to the new environment or social challenge. For instance, intranasal oxytocin increased self-perceived trust only in participants who reported negative mood after social rejection, which was interpreted as motivating social support seeking (Cardoso et al., 2013). Similarly, in the novelty session of the present study, oxytocin, trust and discomfort were mutually positively associated. In contrast, in the familiarity session, trust was associated with calmness and lower oxytocin levels. Oxytocin and trust may facilitate social exposure and confrontation (e.g., in the novelty session), which in turn facilitates acclimatization to social environments (e.g., in the familiarity session).

Stress coping hormones, including oxytocin (e.g., Sutherland et al., 2012), corticotrophin releasing hormone and cortisol (see Schulkin, 2011), are released under novelty conditions, when an object or setting is unfamiliar, dangerous or potentially rewarding. At the same time, social attachment, resources and alliances are implicated in the reduction of stress hormone secretion (Schulkin, 2011). There are only few reports on oxytocin secretion in humans in response to stress or novelty. In one such study, an increase in oxytocin in response to uncontrollable noise was found in women (not in men), and especially in women high on emotionality (Sanders et al., 1990). Taylor et al. (2006) found basal oxytocin levels of postmenopausal women to be higher in those experiencing stress in their relationships with significant partners. A third study found experimentally administered cortisol to increase plasma oxytocin in female subjects. This process was moderated by the subjects' emotionality, with the capacity to express emotions enhancing the oxytocin response to cortisol (Tops et al., 2007b). A challenge that triggered anxiety increased oxytocin in men (Jezova et al., 2013). By contrast, some studies did not find an oxytocin response to a social evaluative stress challenge (Taylor et al., 2006; Cyranowski et al., 2008). However, those last two studies were small and tested lactating or postmenopausal women. By contrast, the only study that tested a larger group of non-lactating, non-postpartum, premenopausal women (and men) found a clear oxytocin response to a social evaluative stress challenge (Pierrehumbert et al., 2010). It is important to note that in this study the stress test was administered during the third visit to the laboratory. This suggests that the subjects had the opportunity to habituate to the social context of the experiment and oxytocin responses to novelty were less likely to mask responses to the stress challenge.

The function of oxytocin in a familiarization-habituation response may also explain results of studies that measured trust behaviorally (e.g., through a “trust game”) or measured oxytocin responses to a task requiring intimate trust (secret sharing). Habituation of autonomic arousal predicted higher oxytocin release during secret sharing, which the authors interpreted as reflecting facilitation of habituation of autonomic arousal and distress responses by oxytocin (Kéri and Kiss, 2011). In a study measuring individual differences in a trust game, higher trust related to lower oxytocin over most of the oxytocin range (Zhong et al., 2012). In that study plasma samples were obtained in a second session a few days after the trust game session, so that oxytocin from the samples may have shown familiarization-habituation effects. Notably, oxytocin may facilitate or consolidate habituation partly during sleep: only women with high oxytocin levels showed an association between support from friends and better sleep quality (Fekete et al., 2013) and sleep was shown to promote inter-session habituation to emotional stimuli (Pace-Schott et al., 2011).

A combination of oxytocin and support appears to be most effective in increasing calmness and decreasing anxiety during stressful social situations (Heinrichs et al., 2003). It has been suggested before that oxytocin may be involved in social aspects of stress coping, i.e., in a “calm and connection” response (Uvnäs-Moberg et al., 2005; cf. Carter, 1998) or a “tend-and-befriend” response (Taylor et al., 2000). In the calm and connection model the oxytocin response is interpreted as a beneficial response to positive social cues, whereas it is less appreciated that oxytocin may be implicated in a stress (coping) response. The interpretation of oxytocin secretion as a tend-and-befriend response is restricted in its scope, as this response is believed to be only displayed by women, and to involve stress-induced motivation to seek support from *already familiar* same-sex friends (Taylor et al., 2000). However, we suggested that the familiarization-habituation response is likely to be a general stress coping response facilitating *familiarization* to novel but permissive social contexts (Tops et al., 2013a,b). The drive to increase familiarity (hypothesized to be stimulated by oxytocin) may of course also stimulate support-seeking from familiar friends. As only young-adult women participated in the current study, future studies need to determine whether men and individuals from different age groups show similar familiarization-habituation responses as compared to young women, and whether such a response may under some conditions facilitate alliance formation (i.e., a tend-and-befriend or “familiarization-habituation-alliance response”). A familiarization-habituation response may be essential in sustaining good health, because a lack of stress response habituation to social contexts would be associated with sustained or repeated physiological costs from stress systems activation, leading to exhaustion and physical health symptoms (Kirschbaum et al., 1995; Kudielka et al., 2006).

Measuring oxytocin from saliva has only recently been validated. Studies have shown that oxytocin levels and ranges were similar in saliva and plasma, concurrently sampled plasma and salivary oxytocin were positively correlated at different time points before and after experimental manipulations, and responded similar to those manipulations (Holt-Lunstad et al., 2008; White-Traut et al., 2009; Grewen et al., 2010; Feldman et al., 2011; Hoffman et al., 2012). For studying the involvement of oxytocin in the familiarization-habituation response the use of salivary oxytocin may be crucial, because it prevents the non-social, physiological stress of venipuncture to obtain plasma samples from disrupting the habituation process. Likewise, the time needed for oxytocin (as well as other hormonal) levels to return to baseline after application of an intravenous catheter may interfere with the novelty of the social context at first blood sampling. Although peripheral oxytocin levels may not reflect central levels and oxytocin mostly does not cross the blood-brain barrier, peripheral oxytocin levels are primarily centrally controlled and there are other routes, such as via the nerves vagus, through which peripheral oxytocin levels feedback upon central processes (see Zhang and Cai, 2011; Churchland and Winkielman, 2012).

Recently, concerns have been formulated that more than only oxytocin molecules are being identified as oxytocin in the current assays (Szeto et al., 2011; McCullough et al., 2013). These other molecules “may represent oxytocin-degradation products and thus may reflect the circulating levels of the peptide, but further studies are needed to evaluate this possibility” (McCullough et al., 2013). Nevertheless, our method (commercial EIA with extraction) is regarded superior to the currently available RIA assays, and to EIA without extraction (Szeto et al., 2011; McCullough et al., 2013). Our method results in oxytocin levels similar to the RIA with extraction used in the 1970s and 1980s (reviewed in McCullough et al., 2013). McCullough et al. (2013) describe these older measurements as independently validated and yielding consistent results in plasma of oxytocin levels <10 pg/mL in healthy adult humans. Despite the need for further validation, our method may be the best available for most researchers who do not have the ability to perform extraction followed by two-dimensional liquid chromatography separation with tandem mass spectrometry detection (as in Zhang et al., 2011). A recent study that applied microdialysis measurement in the brain showed increased oxytocin in extracellular fluid in brain areas (hippocampus and amygdala) in response to intranasal oxytocin, and there were parallel increases in plasma oxytocin, providing evidence that oxytocin reaching behaviorally relevant brain areas is paralleled by changes in plasma oxytocin (Neumann et al., 2013). Intranasal oxytocin also increased oxytocin levels in saliva (Huffmeijer et al., 2012; Van IJzendoorn et al., 2012; Weisman et al., 2012). Although McCullough et al. suggest that increases of oxytocin in saliva after intranasal administration could be the result of “dripping back into the mouth of parts of the sniffs of oxytocin,” this is very unlikely, since, if this is the case, the increase should be strongest directly after intranasal application, rather than peaking at 45–60 min after administration (Weisman et al., 2012).

Reliability of peripheral hormone levels as an individual differences measure is usually determined from the positive association between repeated measures. However, the individuals who displayed high trust in the present study actually showed higher oxytocin levels in the novelty session but lower levels in the familiarity session compared to the individuals who showed lower trust levels. These results suggest that affective, anticipatory and behavioral responses to social context are involved in the regulation of hormone levels (Tops et al., 2006b; Schulkin, 2011), and that such context dependency, and especially the association with novelty and familiarity in repeated measures, should be taken into account when determining reliability (Pruessner et al., 1997). Moreover, the current study illustrates that correlations between trust and oxytocin can either be interpreted in two ways. First, high levels of trust reduce the need for oxytocin induced coping responses, so high trust leads to low levels of oxytocin. Second, high levels of oxytocin facilitate trust, so high oxytocin leads to high levels of trust. These seemingly opposite associations (indicating a negative feedback loop) between oxytocin and trust require authors to be specific about what kind of causal pathway they test/predict^1^.

In conclusion, the present results support the theory that oxytocin is involved in familiarization-habituation responses to novel social contexts or contexts to which the individual did not (yet) successfully acclimatize (Tops et al., 2013a,b). Moreover, we speculate that a history of successful familiarization-habituation responses may generalize to similar or other benign contexts, and may become associated with supportive attachment figures through oxytocin. This may explain the positive associations in the literature between peripheral oxytocin and social distress, partner support and stress coping (e.g., Grewen et al., 2005; Light et al., 2005; Tops et al., 2007a; Holt-Lunstad et al., 2011). The familiarization-habituation response may be essential for maintaining good health by limiting the exposure to physiologically costly stress coping responses.

## Author contributions

Mattie Tops conceived of the study, analyzed the data and wrote the manuscript. Mariëlle Linting analyzed data. Renske Huffmeijer performed the study. All authors reviewed and contributed to the manuscript and contributed to conceiving the study.

### Conflict of interest statement

The authors declare that the research was conducted in the absence of any commercial or financial relationships that could be construed as a potential conflict of interest.

## Footnotes

^1^We thank a reviewer for this suggestion.
